# Experimental Study and Numerical Analysis on the Shear Resistance of Bamboo Fiber Reinforced Steel-Wire-Mesh BFRP Bar Concrete Beams

**DOI:** 10.3390/ma16093446

**Published:** 2023-04-28

**Authors:** Wei Chen, Guohui Qin, Fei Luo, Yuxian Zhu, Gangrui Fu, Siqi Yao, Haohan Ma

**Affiliations:** College of Civil Engineering, Sichuan Agricultural University, Dujiangyan 611830, China; chenwei@sicau.edu.cn (W.C.); 2020326004@stu.sicau.edu.cn (G.Q.); 202108745@stu.sicau.edu.cn (Y.Z.); 202108750@stu.sicau.edu.cn (G.F.); 202108533@stu.sicau.edu.cn (S.Y.); mhh19941108@gmail.com (H.M.)

**Keywords:** bamboo fiber, wire mesh, basalt fiber-reinforced polymer rebar, shear test

## Abstract

Bamboo fiber is a natural and environmentally friendly material made from cheap and widely available resources and is commonly selected as the reinforcement material for steel-wire-mesh BFRPbar concrete beams. In this work, the effects of various fiber lengths and fiber volume rates on the shear properties of bamboo-fiber-reinforced steel-wire-mesh basalt fiber composite reinforcement concrete beams were studied through a combination of shear tests and numerical simulations. The findings demonstrate that the addition of bamboo fiber improves the cracking performance of the beam. The improvement effect of 45 mm bamboo fiber mixed with a 1% volume rate was the most obvious at about 31%. Additionally, the test beam’s total stiffness was increased, and the deflection was decreased. However, the use of bamboo fiber was found to decrease the concrete’s compressive strength, lowering the final shear capacity for the majority of beams. A method for estimating the shear capacity of the bamboo-fiber-reinforced steel-wire-mesh BFRPbar concrete beams is provided and lays the foundation for engineering practice, in accordance with the impact of bamboo fiber and steel wire mesh on beams that suffer shear breaks.

## 1. Introduction

In the construction industry, a basalt fiber-reinforced polymer (BFRP) bar is a high-performance reinforcement material made by pulling, squeezing, and coiling basalt fiber materials and bonding them to the outer surface with epoxy resin. The fiber content of a BFRP bar usually ranges from 75% to 90% with tensile strength between 600 and 1500 MPa. Natural basalt fiber is corrosion resistant, non-toxic, environmentally safe, stable under high temperatures, and has insulating properties. Additionally, steel bars in concrete structures can be replaced with BFRP bars, which solves the corrosion problem [1,2,3,4]. The elastic modulus of a BFRP bar is low with no yield point, so the single application of a BFRP bar will lead to large deformation, increasing cracks, brittle failure, and other problems of concrete members, which needs to be solved urgently [5,6]. Moreover, steel-wire-mesh fine stone concrete is a high-strength composite material with high ductility and toughness [7,8]. Under the application of BFRP bars and steel-wire-mesh composites in concrete, the steel wire mesh lessens deflection and cracking while enhancing the stiffness and bearing capacity of BFRP-bar concrete components [9,10]. When the protective layer thickness of the steel-wire-mesh concrete members is the same as that of the reinforced concrete members, the steel-wire-mesh does not have the desired bending and cracking resistance on members, especially initial cracking resistance [11]. Therefore, the focus of this research paper is how to overcome the unsatisfactory bending and cracking resistance effects of basalt fiber composite reinforcements and steel-wire-mesh acting together in concrete.

Fiber-reinforced concrete has many characteristics, such as strong earthquake resistance, excellent flexural and shear performance and the ability to improve the ductility and toughness of the components [12,13,14,15,16]. The appropriate amount of basalt fibers mixed into concrete produces interpenetrating connections that prevent the development of microcracks and cracks in concrete and change the damage mode of concrete from brittle damage to non-brittle damage; although there is no significant compressive strength, such fibers can effectively improve tensile strength, bending strength, and crack resistance [17,18]. PVA fibers can reduce the occurrence and progression of cracks and increase the crack resistance, but PVA fibers can form a spatial network inside the specimen and prevent air bubbles from being discharged, causing internal defects and reducing compressive strength [19]. Steel fibers can significantly reduce the width and length of cracks and effectively improve the material’s ability to resist crack expansion and increase member toughness [20]. The addition of bamboo fibers to basalt fiber-reinforced concrete beams slows crack initiation and enhances the flexural resistance and rigidity of basalt fiber-reinforced concrete beams [21]. Additionally, short-cut fibers and steel wire mesh can be combined in concrete, and their interaction serves as a complimentary reinforcement that can increase the specimen’s resistance to cracking [11,22].

Bamboo fiber composite materials are strong, wear-resistant, shock absorbing, heat insulating, environmentally friendly, lightweight, energy-saving, and cost-effective. Global forest cover and resource storage have decreased in recent years. However, bamboo area shows a stable growth trend, giving this material undeniably broad development prospects [23]. Consequently, we employ bamboo fibers in this study as the reinforcing material for steel-wire-mesh BFRP-bar concrete beams. By combining experimental research and numerical simulation, we study the shear performance of bamboo-fiber-reinforced steel-wire-mesh BFRP-bar concrete beams and analyze the shear performance of different lengths and different volume rates of mixed bamboo fiber on the beams based on the optimal length and volume rate.

## 2. Materials and Methods

### 2.1. Materials

The test was conducted using concrete with a standard compressive strength of 40 MPa in cubic blocks and a strength rating of 42.5 for regular silicate cement. For coarse-grain material, we selected 5~10 mm continuous-graded gravel to facilitate passage through the wire mesh, improving the performance of the concrete. Medium sand was selected as the fine-grain material, as shown in Table 1. Typical long and thin rod-type bamboo was selected as the fiber and was made from natural bamboo material cut by a machine, with relevant parameters shown in Table 2. The wire mesh had a diameter of 1 mm and a mesh size of 10 mm × 10 mm. This member was reinforced longitudinally with BFRP bars 20 mm in diameter. Table 3 lists the relevant mechanical properties. The hoop and erector bars were hot-rolled steel bars with a diameter of 8 mm and a standard tensile strength of 300 MPa.

### 2.2. Test Beam Design

**Figure 1:**
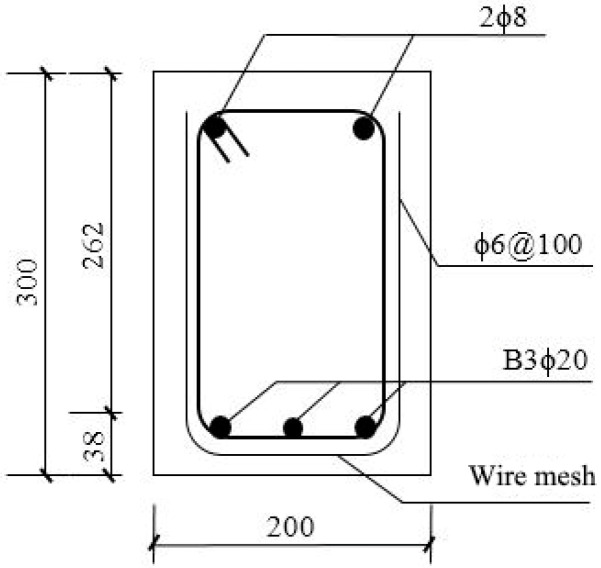
Cross-sectional reinforcement drawing.

**Figure 2:**
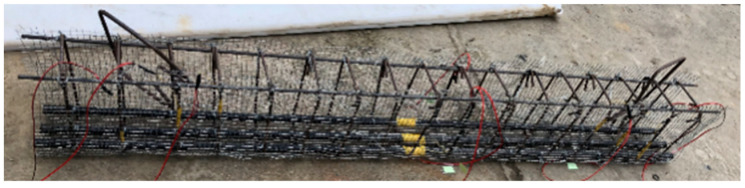
Schematic diagram of reinforcement framework.

The test was designed with eight test beams using the fiber length and fiber volume rate as variation parameters, as shown in Table 4. The design size of each beam was b × h × l = 200 mm × 300 mm × 1800 mm, and the effective cross-sectional height was h_0_ = 262 mm. To achieve shear failure of the test beam’s intended purpose, the test beam was reinforced longitudinally using three BFRP bars 20 mm in diameter, and two pieces of hot-rolled steel bars with a standard tensile strength of 300 MPa and a diameter of 8 mm were provided near the top of the beam as erection bars. The hot-rolled steel bars used for the hoops were 8 mm in diameter with a specified tensile strength of 300 MPa. They were evenly spaced at a distance of 100 mm along the whole beam. The section reinforcement is shown in Figure 1. As seen in Figure 2, all test beams were set up with wire mesh at the full beam.

### 2.3. Measuring Point Layout and Loading Scheme

Steel strain gauges were arranged in the middle of the span of the three basalt fiber composite bars at the bottom, as well as in the shear span area where the line between the support and the loading point intersected with the corresponding hoop reinforcement. The concrete strain gauge was arranged at the connection between the shear span support at both ends of each beam and the loading point. A DH3818Y strain tester was used to collect strain data. For monitoring the overall deformation of the beam, a displacement meter was placed in the middle span above the support and below the loading point.

**Figure 3:**
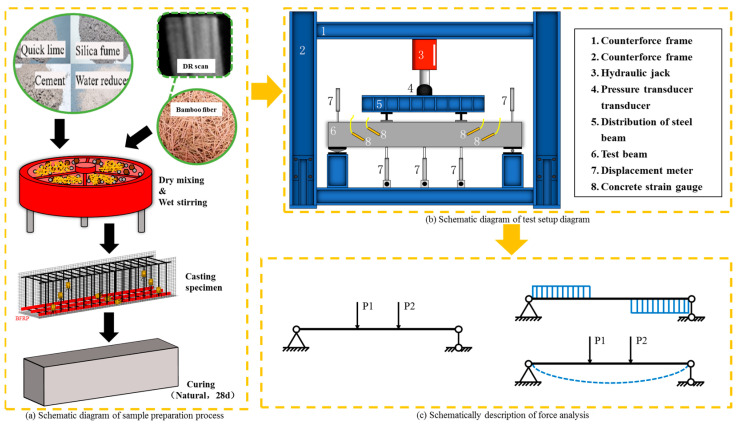
Experimental flow chart [21].

The test loading apparatus was chosen to be a model ZHP-300B 500 kN uniaxial electro-hydraulic servo loading system, as illustrated in Figure 3. Loading was carried out in stages. Before the oblique section cracked, each stage load was 5 kN, and the load was held for 5 min. When the load approached 90% of the inclined section’s predicted cracking load, the load was 2.5 kN per level, and the load holding time was extended to 10 min until the first inclined crack appeared. After the oblique section cracked, the load difference of each stage was increased to 10 kN and the load holding time was 5 min. In order to overload until failure, the displacement control method was used when the load accumulated up to 90% of the anticipated maximum load. During the loading process, a crack width measuring instrument was used to record crack development.

## 3. Experimental Results and Discussion

### 3.1. Damage of the Test Beam

**Figure 4:**
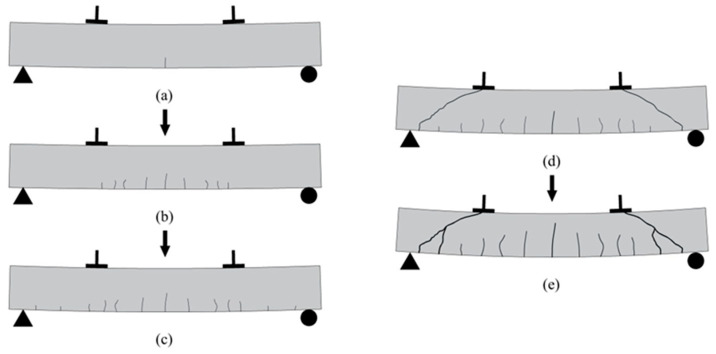
Schematic diagram of beam crack development: (**a**) vertical bending cracks; (**b**) cracks appeared in the pure bending zone; (**c**) cracks appeared towards the bearing position; (**d**) flex–shear inclined cracks; (**e**) critical oblique crack.

**Figure 5:**
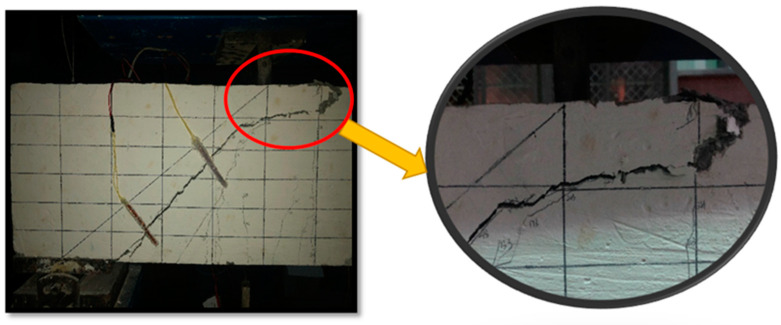
Shear cross section frontal damage and concrete is crushed at the loading point.

All eight test beams presented shear compression damage. In the process of graded loading, under loading to the ultimate bearing capacity of 9~16% of the span in the tensioned area, there first appeared small vertical bending cracks (as seen in Figure 4a). Then, the width and height of vertical cracks slowly increased, and more cracks appeared in the pure bending zone, spreading from mid-span to shear span (as seen in Figure 4b). When the load increased to 18~25% of the ultimate bearing capacity, cracks continuously appeared towards the bearing position (as seen in Figure 4c). When the load was between 27% and 33% of the maximum carrying capacity, the vertical cracks near the support extended upward obliquely to form flex–shear inclined cracks. Meanwhile, the vertical cracks in the pure curved zone began to develop slowly and some even stopped (as seen in Figure 4d). As the load increased, oblique cracks continued to grow in number and width, extending towards the loading point. The broadest primary oblique crack, called the critical oblique crack, was formed between the loading point and the support (as seen in Figure 4e). By continuing to increase the load to between 44% and 53% of the ultimate bearing capacity, the hoop yielded in the bending and shearing section, followed by rapid development of the critical oblique fracture width. Part of the concrete near the loading point was crushed, and the bearing capacity could not continue to increase, resulting in failure of the component. The test diagram shows the damage condition of the L-45-1 oblique section shown in Figure 5.

### 3.2. Test Result

Table 5 displays each specimen’s mechanical characteristics, shear cracking load, and shear ultimate bearing capacity (*f_cu_* is cubic compressive strength, and *f_t_* is cubic splitting tensile strength).

### 3.3. Analysis of Test Results

#### 3.3.1. Cracking Resistance Analysis

**Figure 6:**
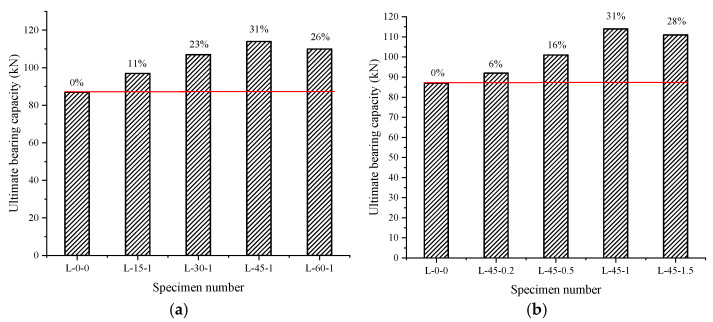
Cracking load of the specimen: (**a**) Relationship between fiber length and cracking load; (**b**) relationship between fiber volume rate and cracking load.

To a certain extent, the inclusion of bamboo fiber was found to improve the cracking load of steel-wire-mesh BFRP-bar concrete beams compared to L-0-0, as illustrated in Figure 6, because bamboo fiber was randomly distributed in the concrete specimens. By bearing stress like a steel bar, the bamboo fiber increased the effective restraint area of longitudinal reinforcement, serving as a “bridge frame”, inhibiting the development of cracks, and increasing the tensile strength of the fiber concrete. Furthermore, the bamboo fibers distributed in the shear span area acted as miniature stirrups and bore part of the shear force, improving the crack resistance of the inclined section of the test beam. Among them, the L-45-1 beam presented the most obvious lifting effect, with an increase of 31%. As shown in Table 5, this increase occurred due to the L-45-1 concrete having higher tensile strength.

Comparing specimens L-15-1, L-30-1, L-45-1, and L-60-1 (as seen in Figure 6a) demonstrates that the cracking load of specimens with fiber lengths under 45 mm increased as the length of the fiber decreased due to the strong bond between the bamboo fibers and concrete: The longer the bamboo fiber portion of the bond, the better the bond effect between the two, and the greater the ability to inhibit the development of cracks. However, the cracking load of 60 mm fiber-length specimens was lower than 45 mm because there was interaction between the bamboo fiber and steel-wire-mesh. When the length of the fiber was longer than the steel-wire-mesh’s mesh size, the mesh partially prevented passage of the bamboo fiber because it was attached to the steel-wire-mesh. This caused the material distribution inside the component to be uneven, leading to internal flaws and different mechanical properties in different distribution areas. At the same time, the longer the fiber, the easier it was able to form local defects inside the concrete. For these two reasons, the lifting effect of the fiber-reinforced material was not achieved.

Comparing specimens L-45-0.2, L-45-0.5, L-45-1, and L-45-1.5 (as seen in Figure 6b) shows that the cracking loads of specimens with fiber volume percentages within 1% increased as the fraction of fiber volume increased, as a larger fiber volume percentage enabled more fiber content to act as miniature reinforcements, better inhibiting the development of cracks. The cracking load of the specimens with 1.5% fiber volume ratio was lower than that with a 1% fiber volume ratio because an excessive fiber volume ratio enabled local defects to readily form on the specimen. At the same time, an excessive volume ratio was more likely to yield an interaction between the bamboo fiber and steel wire mesh. These two reasons together led to a decrease in the crack resistance of specimen L-45-1.5.

#### 3.3.2. Analysis of Ultimate Shear Capacity

**Figure 7:**
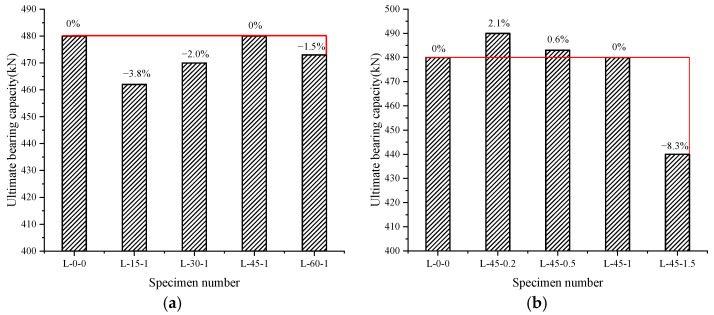
Comparison of the ultimate load of each specimen. (**a**) The relationship between fiber length and ultimate load; (**b**) the relationship between fiber volume rate and ultimate load.

According to Figure 7, the addition of bamboo fiber somewhat reduced the ultimate bearing capacity of the steel-wire-mesh BFRP-bar concrete beams compared to the steel-wire-mesh BFRP-bar concrete beams L-0-0, with the exception of beams L-45-0.2, L-45-0.5, and L-45-1. This result occurred because the final bearing capacity of the test beam was established by concrete being crushed close to the shear crossing loading point, demonstrating the critical function of concrete’s compressive strength. When bamboo fiber was mixed into concrete at a certain volume rate, it replaced a portion of the concrete aggregate, reducing the aggregate ratio of the whole concrete specimen. However, the bearing capacity of bamboo decreased to a certain extent compared to that of concrete. Thus, the whole specimen’s compressive strength value decreased, leading the ultimate bearing capacity of the specimen to decrease accordingly. The beam L-45-1.5 dropped the most (8.3%), as seen in Table 5, because the compressive strength of the concrete in L-45-1.5 was lower.

Figure 7a shows that the ultimate bearing capacity of specimens with fiber lengths less than 45 mm improved with an increase in fiber length, based on a comparison of specimens L-15-1, L-30-1, L-45-1, and L-60-1, because the compressive strength of the concrete affected the shear performance of the specimen. The longer the fiber length, the better the bonding effect, and the higher the compressive strength (as seen in Table 5), the higher the ultimate shear capacity. However, in contrast to the specimen with a 45 mm fiber length, the 60 mm specimen’s maximum bearing capacity was lower. This result was again due to the excessive fiber length leading to a decrease in crack resistance.

Comparing specimens L-45-0.2, L-45-0.5, L-45-1, and L-45-1.5 (as seen in Figure 7b) demonstrates that when the fiber length remained the same, the ultimate bearing capacity of the specimens diminished as the fraction of fiber volume increased because the compressive strength of the bamboo fiber concrete test blocks decreased with an increase in the fiber volume percentage (as shown by *f_cu_* in Table 5). At the same time, it can be seen that the decrease in specimens with a 1.5% fiber volume percentage was more pronounced. Again, the reason for this result is that the excessive fiber volume percentage led to a decrease in crack resistance.

#### 3.3.3. Deflection Analysis

**Figure 8:**
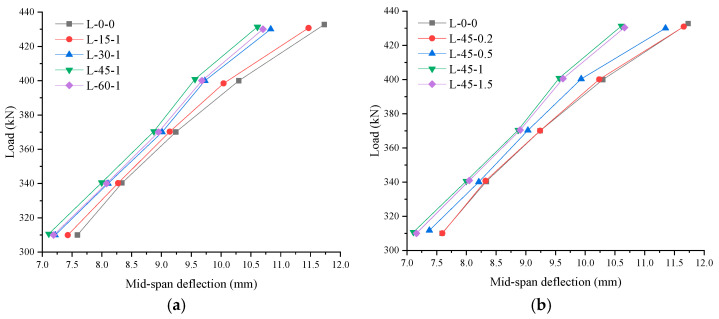
Load–deflection relationship diagram of the test beam: (**a**) Relationship between fiber length and deflection; (**b**) relationship between fiber volume rate and deflection.

Figure 8 illustrates the load–deflection curve for each test beam. Each specimen’s deflection under the same stress prior to breaking was not significantly different. Here, the load–deflection curve presents a linear development. All test beams are in the elastic deformation stage, in which the deflection of the members is not significantly affected by the inclusion of bamboo fiber. After concrete cracking, the ultimate bearing capacity is the elastic–plastic stage, in which the development of deflection is more obvious. Under the same load, the deflection of specimens mixed with bamboo fiber decreased, and the deflection gap became more obvious with an increase in the load. Bamboo fiber offers excellent cracking resistance, which can effectively prevent the further spread of cracks, jointly bear tensile stress with concrete, and reduce the stress of the specimen at the crack points of BFRP bars. With the inclusion of these fibers, the whole specimen’s stiffness improved, and the deflection after being mixed into concrete was reduced. When the specimen reached its maximum bearing capacity, it entered the failure stage, during which the residual bearing capacity steadily declined, clearly demonstrating brittleness. Additionally, the residual bearing capacity diminished as the deflection increased. In addition, the decline rate of the residual bearing capacity of the specimen with the addition of bamboo fiber was lower than that without bamboo fiber. Clearly, adding bamboo fiber improved the component’s overall deformation capacity. The concrete beam L-45-1 presented the most obvious lifting effect.

Figure 8a shows that the deflection of specimens with fiber lengths less than 45 mm reduced with an increase in fiber length based on a comparison of specimens L-15-1, L-30-1, L-45-1, and L-60-1, because longer fiber lengths increased the specimen’s tensile strength, allowing it to withstand tensile stress in the concrete and decrease specimen deflection. The deflection of the specimen with a 60 mm fiber length was lower than that of the 45 mm one. Again, the excessive length of the fiber led to a decrease in the crack resistance.

Comparing specimens L-45-0.2, L-45-0.5, L-45-1, and L-45-1.5 (as depicted in Figure 8b) clearly shows that the deflection of specimens with fiber volume percentages within 1% decreased with an increase in fiber volume percentage. This result occurred because when more bamboo fiber was added, the bamboo fiber was able to better withstand the tensile stress in the concrete, thereby lowering specimen deflection. However, specimens with a 1.5% fiber volume percentage deflected less stress than specimens with a 1% fiber volume percentage—again, because too much fiber yielded a reduction crack resistance.

## 4. Numerical Simulation

### 4.1. Numerical Simulation of Beams

#### 4.1.1. Constitutive Relationships of Materials

A plastic damage model was used for bamboo fiber concrete. In addition, an integral modeling form was adopted to disperse bamboo fibers into the concrete as a whole and as a continuous homogeneous material. A reference was used for the constitutive relationship of bamboo fiber concrete [24]. The “Code for Design of Concrete Structures” (GB50010-2010) Appendix C’s uniaxial constitutive relation was adopted [25].

**Figure 9:**
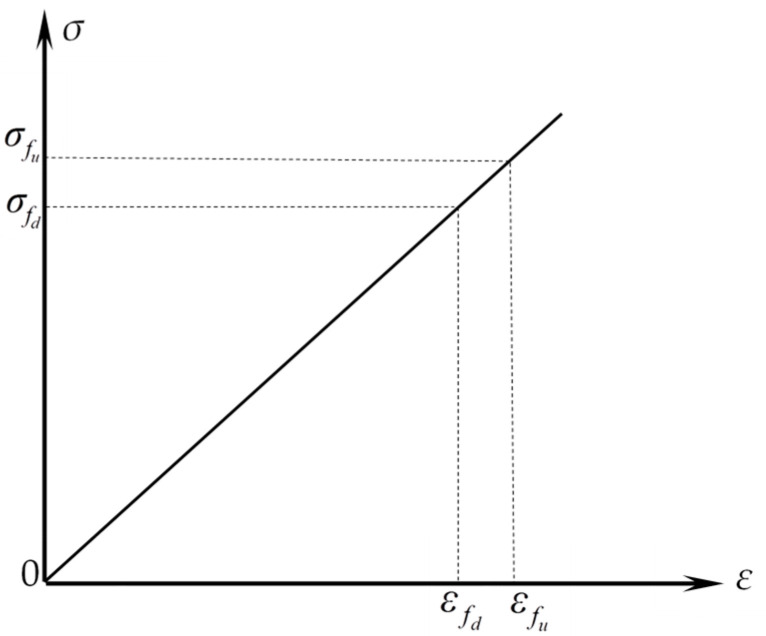
Stress–strain relationship of the BRFP bars.

Figure 9 depicts the stress–strain connection of BFRP bars [26], and Equation (1) depicts the function relationship.

(1) $$\sigma_{f}=E_{f}\varepsilon_{f}\left(0\leq \varepsilon_{f}\leq \varepsilon_{f_{u}}\right)$$

where: $\sigma_{f}$ is the BFRP bars’ stress; $\varepsilon_{f}$ is the BFRP bars’ strain; $E_{f}$ is the BFRP bars’ elastic modulus; and $\varepsilon_{f_{u}}$ is the BFRP bars’ ultimate tensile strain.

**Figure 10:**
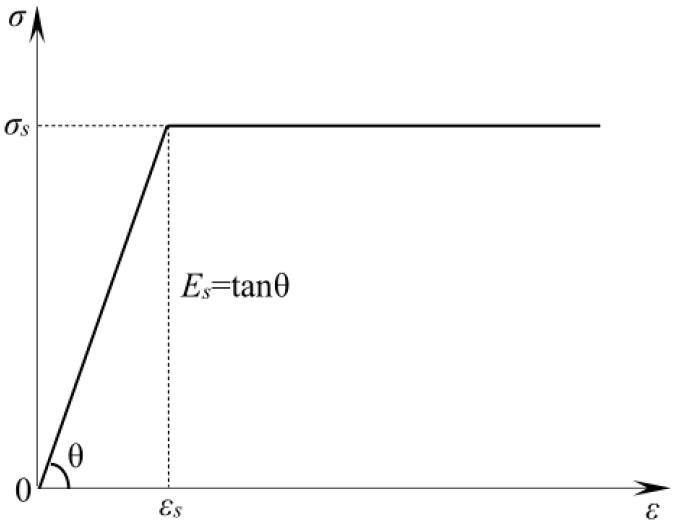
Stress–strain relationship of steel.

In the analysis, various types of steel, such as reinforcements, steel wire mesh, and additional metal mats, were considered as isotropic ideal elastic–plastic materials. Their stress–strain relationships are shown in Figure 10, and their functions are shown in Equations (2) and (3):

(2) $$\sigma_{s}=E_{s}\cdot \varepsilon_{s}\left(\varepsilon \leq \varepsilon_{s}\right)$$

(3) $$\sigma =\sigma_{s}\left(\varepsilon >\varepsilon_{s}\right)$$

where: $\sigma_{s}$ is the steel stress; $\varepsilon_{s}$ is the steel strain; and $E_{s}$ is the steel elastic modulus.

#### 4.1.2. Model Building

**Figure 11:**
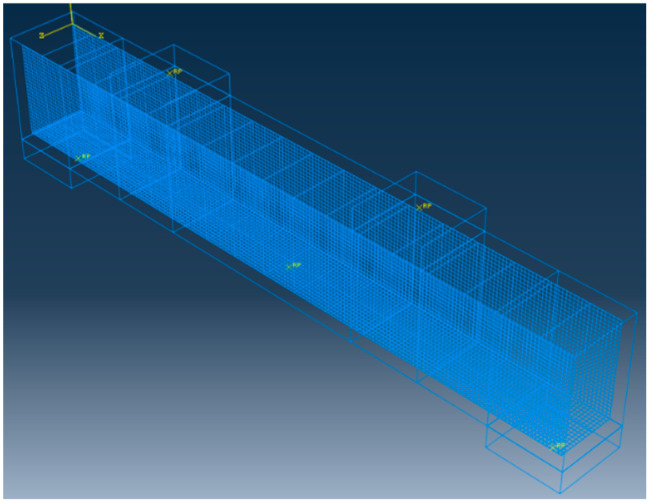
Finite element model.

**Figure 12:**
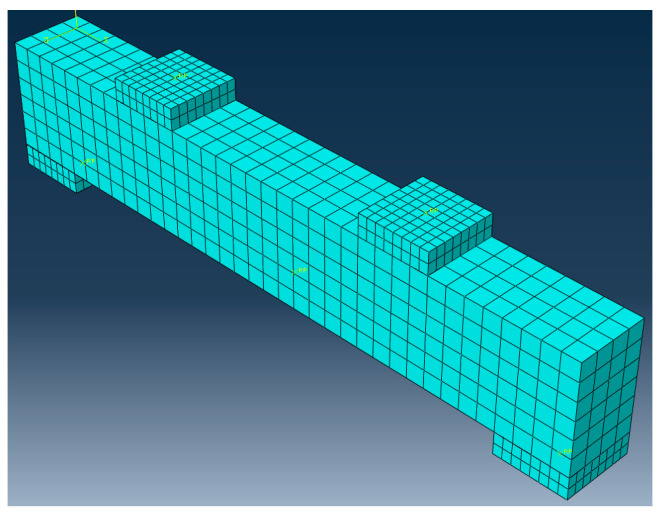
Mesh generation.

Figure 11 shows the finite element model of bamboo fiber reinforced steel-wire-mesh BFRP-bar concrete beams. During the model building process, a rigid mat under the centralized force loading of the concrete beam and at the support and a three-dimensional eight-node linear reduced integration unit (C3D8R) were used. The BFRP bar and erection bars, hoop bars, and steel wire mesh all use two-node three-dimensional linear truss units (T3D2). By embedding them into concrete, BFRPbars, erection bars, hoop bars, and steel-wire-mesh were joined to form a complete steel skeleton that already exists, allowing for interaction effects. To prevent stress concentration, a tie mode connection was made between the rigid plate and concrete beam. A reference point was established on both the support and the rigid pad at the loading point, and then the reference point was coupled with the surface of the rigid pad via coupling so that the boundary conditions set on the reference point could be applied to the model beam. The constraints of X, Y, and Z = 0 correspond to the reference point of one end of support on the simulated beam, while Y and Z = 0 correspond to the reference point at the other end of the support. These constraints transform the simulated beam into a simply supported beam. The displacement loading approach was used in this research as a control. The concrete unit has a 50 mm grid size, as shown in Figure 12.

### 4.2. Analysis of Numerical Results

**Figure 13:**
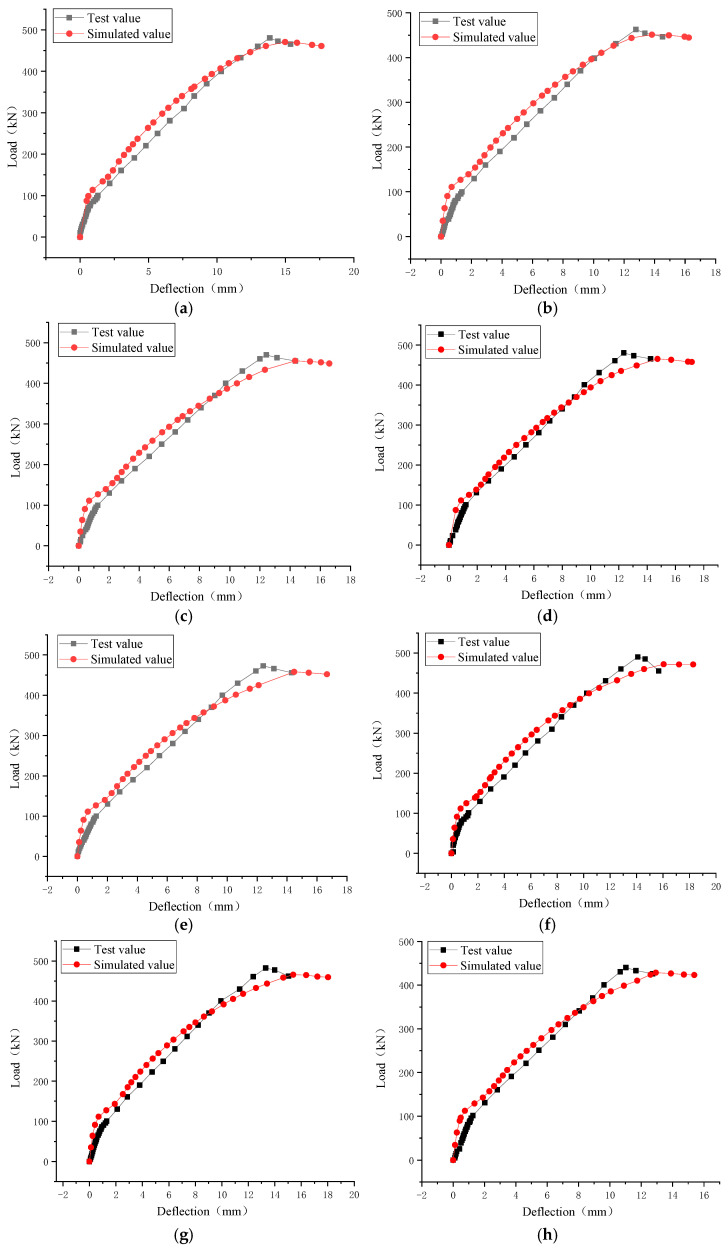
Comparison of the tested and simulated values of the beam load–deflection curve: (**a**) L-0-0; (**b**) L-15-1; (**c**) L-30-1; (**d**) L-45-1; (**e**) L-60-1; (**f**) L-45-0.2; (**g**) L-45-0.5; (**h**) L-45-1.5.

Figure 13 provides comparison pictures of the load–deflection curve obtained via numerical simulation and the load–deflection curve obtained through testing. Based on the slope of the deformation curves for the two values, the stiffness of the simulated value was marginally higher than that of the test value in the elastic stage. Once it enters the working stage, the simulated value’s trend matches that of the test value. Here, the simulated value rapidly approaches the failure point, although there is no significant variation in shear ultimate capacity. Table 6 presents a comparison of the experimental and predicted data for each beam’s shear cracking load and shear ultimate capacity.

In Table 6, the ratio of the test value to the simulated value of the shear cracking load has an average value, standard deviation, and coefficient of variation of 1.03, 0.113, and 0.11, respectively. The ratio between the experimental value and the simulated value has an average value of 1.03, a standard deviation of 0.007, and a coefficient of variation of 0.007. These data indicate that there is good agreement between the experimental findings and numerical simulation of this model. This paper explains the rationality and correctness of the selection of the numerical simulation model. As a result, it is now possible to utilize the Abaqus finite element software to study the shear performance of bamboo fiber reinforced steel-wire-mesh BFRP-bar concrete beams, opening up a practical route for the adoption and popularization of this class of concrete buildings.

## 5. Shear Capacity

### 5.1. Theoretical Calculation Method for the Shear Capacity of Oblique Section

There are many factors affecting the shear capacity of the flexural components, and there are reciprocal effects between the factors and the materials because the shear failure process of reinforced concrete beam is complicated. Therefore, it is very difficult to accurately anticipate the flexural member’s shear capacity. In this test, there were many differences observed between the test beam and the ordinary reinforced concrete beam. In addition to taking into account how the short cut bamboo fiber’s bridging action affects shear resistance, one must also consider how the steel-wire-mesh binding on the reinforced skeleton improves shear resistance, which complicates the shear load inside the flexural member.

Based on existing research results on shear capacity, we consider the influence of BFRP bars, bamboo fibers and steel-wire-mesh added to concrete against shear capacity. We developed an empirical formula for the shear capacity of bamboo fiber reinforced steel-wire-mesh BFRP-bar concrete beams by fitting, taking into account the shear capacity calculation formula in [27,28,29,30,31,32,33,34,35,36] and the available test data:

(4) $$V_{u}=V_{sv}+V_{smv}+V_{fc}$$

where: $V_{u}$ is the oblique shear capacity of bamboo fiber reinforced steel-wire-mesh BFRP-bar concrete beams; $V_{sv}$ is the stirrup’s shear bearing capability; $V_{smv}$ is the shear capacity of vertical steel wire; and $V_{fc}$ is the oblique shear resistance of fiber-reinforced concrete beams without belly reinforcing.

The shear capacity of stirrups (*V_sv_*) is given by GB50010-2010 “Code for Design of Concrete Structures” [25] as follows:

(5) $$V_{sv}=f_{yv}\frac{A_{sv}}{s}h_{0}$$

where: $f_{yv}$ is the stirrup’s tensile strength design value; $A_{sv}$ is the same section inside the stirrup section area; s is the distance between stirrups along the member’s length; and $h_{0}$ represents the section’s effective height.

As can be seen from the section reinforcement diagram in Figure 1 that the steel-wire-mesh is arranged as a “u” shape in the cross section of the reinforcement skeleton of the test beam. The mechanism is the same as that of the stirrup, which can improve the shear capacity to a certain extent. The shear capacity *V_smv_* of vertical steel wire can be calculated as follows:

(6) $$V_{smv}=f_{smv}\frac{A_{smv}}{s_{mv}}h_{0}$$

where: $f_{smv}$ is the vertical steel wire tensile strength design value; $A_{smv}$ is the steel wire’s section area in the same section; and $s_{mv}$ is the spacing between vertical steel wires along the length direction of the member.

In both the domestic and international literature, various formulae have been proposed for determining the shear capacity of different fiber-reinforced concrete beams. The equation for determining a fiber-reinforced concrete beam’s shear capacity on an oblique section is given in the Chinese “Technical Specification for Fiber-Reinforced Concrete Structures” CECS38:2004 [27]. This equation demonstrates that adding short-cut fibers can increase the concrete specimens’ tensile strength, further enhancing the beams’ shear characteristics. Therefore, when calculating the shear capacity *V*_fc_ of an inclined section of fiber-reinforced concrete beams, the fiber influence coefficient *β_v_* and fiber characteristic parameter *λ_f_* should be referred to based on the concrete’s shear capacity *V*_c_ [28]:

(7) $$V_{fc}=V_{c}\left(1+\beta_{v}\lambda_{f}\right)$$

where: $V_{c}$ is the concrete beam’s oblique shear capacity without belly reinforcement; $\beta_{v}$ is the fiber’s effect coefficient on the concrete beam’s inclined section’s shear capacity, which is determined by the test results; $\lambda_{f}$ is the fiber characteristic parameter, $\lambda_{f}=p_{f}\times \frac{l_{f}}{d_{f}}$; $p_{f}$ is the volume fraction of fibers; $l_{f}$ represents the fiber length; and $d_{f}$ represents the fiber diameter.

The longitudinal tensile steel bars are high-performance FRP bars made of basalt fiber, which have a different influence than ordinary steel bars on the shear capacity of the flexural members. The calculation formula for the inclined section shear capacity of FRP-bar concrete beams without abdominal bars, given by our specification GB50608-2010 “Application Technical Specification for Construction of Fiber Reinforced Composite Materials” [29], is as follows:

(8) $$V_{c}=0.86f_{t}bh_{0}\left(\sqrt{2p_{fl}n_{f}+{\left(p_{fl}n_{f}\right)}^{2}}-p_{fl}n_{f}\right)$$

where: $f_{t}$ is the concrete axis’s designed tensile strength; b represents the rectangular section’s width; $p_{fl}$ is the ratio of longitudinal tensile FRP bars; $p_{fl}=\frac{A_{f}}{bh_{0}}$; $A_{f}$ is the longitudinally stretched FRP bars’ section area; $n_{f}$ is the proportion of the elastic modulus of concrete to that of FRP bars, $n_{f}=\frac{E_{f}}{E_{c}}$; $E_{f}$ is the FRP bars’ elastic modulus; and $E_{c}$ is the elastic modulus of concrete.

By substituting Formulas (8) into (7) and then substituting (5), (6), and (7) into (4), the final calculation formula of the oblique section shear capacity *V*_u_ of the bamboo fiber reinforced steel-wire-mesh BFRP-bar concrete beam can be obtained as follows:

(9) $$V_{u}=\left[\begin{array}{l} 0.86f_{t}b\left(\sqrt{2\rho_{fl}n_{f}+{\left(\rho_{fl}n_{f}\right)}^{2}}-\rho_{fl}n_{f}\right)\\ \left(1+\beta_{v}\lambda_{f}\right)+f_{smv}\frac{A_{smv}}{s_{mv}}+f_{yv}\frac{A_{sv}}{s} \end{array}\right]h_{0}$$

**Figure 14:**
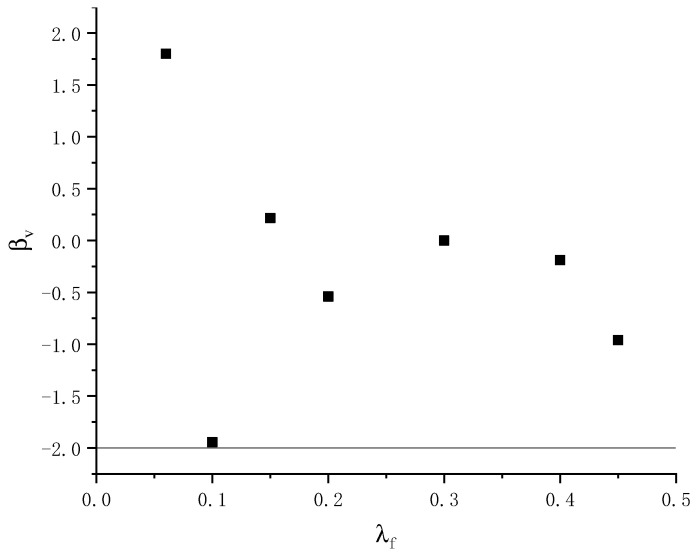
Relationship curves of λ*_f_* and *β*_v_.

The coordinate system was established with *λ_f_* as the horizontal axis and *β_v_* as the vertical axis. The corresponding values of *λ_f_* and *β_v_* when the oblique section of each specimen was damaged by shear were placed in the coordinate system, as shown in Figure 14. Considering structural security, *β_v_* = −1.972 under the packet line can be determined as the *β_v_* value.

### 5.2. Comparative Analysis between the Tested and Theoretical Values

A comparison of the experimental and theoretical results of the shear ultimate bearing capacity of each test beam is presented in Table 7.

The ratio between the test value and estimated value, as shown in Table 7, has an average value of 1.14, a standard deviation of 0.035, and a coefficient of variation of 0.031. This result demonstrates that the calculation results of the calculation method are in good agreement with the actual test data and can serve as the basis for calculating the inclined section shear capacity of bamboo-fiber-reinforced steel-wire-mesh BFRP-bar concrete beams.

## 6. Conclusions

This study examined concrete beams made of bamboo-fiber-reinforced steel-wire-mesh BFRP bar using shear testing and numerical modeling. The conclusions are as follows:

(1) After the bamboo fiber is added to the concrete, it functions as a reinforcement able to withstand tensile stress alongside the concrete, thereby postponing the onset of the concrete beam’s initial crack and enhancing cracking load. The improvement effect of 45 mm bamboo fiber mixed with a 1% volume rate was the most obvious at about 31%.
(2) The inclusion of bamboo fiber can stop the crack from spreading further, lower the specimen’s stress at the crack point on the BFRP bar, and increase the beam’s overall stiffness, all of which work to lessen the deflection of the beam.
(3) As bamboo fiber reduces the compressive strength of fiber-reinforced concrete, it has a slightly negative impact on the ultimate load of the beams. The largest drop, roughly 8.3%, was observed under a 1.5% volume ratio with the 45 mm bamboo fiber beam.
(4) The findings of the numerical simulation are essentially in line with the outcomes of the experiments, which validates the accuracy of the numerical analysis methodology and establishes a basis for the simulation study of bamboo-fiber-reinforced steel-wire-mesh BFRP-bar concrete beams.
(5) A formula was established to determine the shear load capacity of the bamboo-fiber-reinforced steel-wire-mesh BFRP bar and concrete beam oblique sections. The shear capacity of this type of member can be accurately predicted using this formula, which is straightforward in form and has excellent agreement with the experimental results. This formula also serves as a theoretical foundation for actual engineering work.

## 7. Outlook

Based on this paper, a few areas remain to be studied in depth:

(1) This study only considered long thin rod-type bamboo fibers. However, the bonding effects between different fiber shapes and substrate may be different, so more bamboo fiber shapes, such as bamboo filament types, bamboo piece types, and other shapes should be considered for future research.
(2) Although plant fiber material is easy to obtain, it is also easy for it to become corroded in the concrete environment, so future research should be carried out based on two aspects: the processing of bamboo fiber surface and the modification of cement.
(3) Wire mesh was woven from vertical and longitudinal wires, and the vertical wire portion more obviously improved shear performance. The effect of the wire mesh as a variable on the shear performance of the test beam, however, was not considered in this paper, so the improvement effects on shear performance after changing the size and material of the wire mesh could be considered in future work.

## Figures and Tables

**Table 1:** Concrete mix ratio.

Water (kg/m^3^)	Cement (kg/m^3^)	Crushed Stone (kg/m^3^)	Medium Sand (kg/m^3^)
215	500	1095	590

**Table 2:** Performance parameters of the bamboo fiber.

Material	Fiber Diameter (mm)	Fiber Length (mm)	Density (kg/m^3^)
Bamboo fiber	1.5	15/30/45/60	848.826

**Table 3:** Mechanical properties of the BFRP bar.

Diameter (mm)	Tensile Strength (MPa)	Modulus of Elasticity (GPa)
20	1010.77	44

**Table 4:** Parameters of the test beam.

Specimen	Bamboo Fiber Length (mm)	Bamboo Fiber Volume Percentage (%)
L-0-0	0	0
L-15-1	15	1
L-30-1	30	1
L-45-1	45	1
L-60-1	60	1
L-45-0.2	45	0.2
L-45-0.5	45	0.5
L-45-1.5	45	1.5

**Table 5:** Main test data.

Specimen	*f_cu_* (MPa)	*f_t_* (MPa)	Cracking Load (kN)	Ultimate Load (kN)
L-0-0	47.6	3.01	87	480
L-15-1	44.9	3.13	97	462
L-30-1	45.4	3.59	107	470
L-45-1	46.2	3.75	114	480
L-60-1	45.7	3.64	110	473
L-45-0.2	48.3	3.04	92	490
L-45-0.5	46.8	3.23	101	483
L-45-1.5	41.2	3.67	111	440

**Table 6:** Comparison between the test results and model simulation results.

Specimen	Shear Cracking Load		Test Value/Simulation Value	Ultimate Shear Capacity		Test Value/Simulation Value
	Test Value (kN)	Simulated Value (kN)		Test Value (kN)	Simulated Value (kN)	
L-0-0	87	102	0.85	240	235	1.02
L-15-1	97	97	1	231	225.5	1.02
L-30-1	107	98	1.09	235	227.5	1.03
L-45-1	114	101	1.13	240	232.5	1.03
L-60-1	110	99	1.11	236.5	229	1.03
L-45-0.2	92	104	0.88	245	236	1.04
L-45-0.5	101	101	1	241.5	233	1.04
L-45-1.5	111	93	1.19	220	214	1.03
Average value			1.03			1.03
Standard deviation			0.113			0.007
Coefficient of variation			0.110			0.007

**Table 7:** Analysis of test data and theoretical shear ultimate bearing capacities for comparison.

Specimen	$\mathrm{Test}\;\mathrm{Value}\;V_{u}^{t}\;(\mathrm{kN})$	$\mathrm{Simulated}\;\mathrm{Value}\;V_{u}^{c}\;(\mathrm{kN})$	$V_{u}^{t}/V_{u}^{c}$
L-0-0	240	217.5	1.10
L-15-1	231	212.9	1.08
L-30-1	235	208.2	1.13
L-45-1	240	203.6	1.17
L-60-1	236.5	199	1.19
L-45-0.2	245	214.7	1.14
L-45-0.5	241.5	210.6	1.15
L-45-1.5	220	196.7	1.12
Average value			1.14
Standard deviation			0.035
Coefficient of variation			0.031

Note: The measured value of shear bearing capacity $V_{u}^{t}$ can be obtained by calculating the beam shear force in material mechanics.

## Data Availability

The data presented in this study are available on request from the corresponding author.
